# Crystal structure, Hirshfeld surface analysis of 2-(eth­oxy­carbon­yl)quinolinium tetra­chlorido(quinoline-2-carboxyl­ato-κ^2^*N*,*O*)stannate(IV) monohydrate

**DOI:** 10.1107/S2056989026003932

**Published:** 2026-04-29

**Authors:** Tarek Benlatreche, Boutheina Boualia, Mohamed Abdellatif Bensegueni, Stéphane Golhen

**Affiliations:** a Environmental and Structural Molecular Chemistry Research Unit URCHEMS Faculty of Exact Sciences University of Constantine 1-Mentouri Brothers 25000 Algeria; b National Higher School for Hydraulics, Abdellah Arbaoui, Blida, Algeria; c CNRS Rennes Institute of Chemical Sciences -UMR 6226 University of Rennes France; Venezuelan Institute of Scientific Research, Venezuela

**Keywords:** crystal structure, Hirshfeld surface analysis, C—H⋯O hydrogen bond, 2-(eth­oxy­carbon­yl)quinolinium cation, tin(IV)

## Abstract

The asymmetric unit of the title hydrated complex salt, (C_12_H_12_NO_2_)[Sn(C_10_H_6_NO_2_)Cl_4_]·H_2_O, consists of one 2-(eth­oxy­carbon­yl)quinolinium cation, one tetra­chlorido­(quinolinium-2-carboxyl­ato)stannate(IV) anion and one water mol­ecule. The compound was obtained by reaction of quinaldic acid with tin(II) chloride dihydrate in ethanol. The Sn^IV^ atom is six-coordinated by four chloride ligands and by one N and one O atom from the quinolinium-2-carboxyl­ate ligand, forming a distorted octa­hedral coordination environment. In the mol­ecular structure, intra­molecular O—H⋯O and C—H⋯Cl hydrogen bonds are observed. In the crystal, N—H⋯O, C—H⋯O and C—H⋯Cl hydrogen bonds link the components into a three-dimensional network. In addition, Y—*X*⋯π (Sn—Cl⋯π) and π–π stacking inter­actions involving three aromatic rings are present, with centroid–centroid separations in the range 3.633 (2)–3.864 (2) Å.

## Abstract 2

The asymmetric unit of the title hydrated complex salt, (C_12_H_12_NO_2_)[Sn(C_10_H_6_NO_2_)Cl_4_]·H_2_O, consists of one 2-(eth­oxy­carbon­yl)quinolinium cation, one tetra­chlorido­(quinolinium-2-carboxyl­ato)stannate(IV) anion and one water mol­ecule. The compound was obtained by reaction of quinaldic acid with tin(II) chloride dihydrate in ethanol.

## 1. Chemical context

Quinolinium derivatives bearing carboxyl­ate groups are attractive ligands because they combine an aromatic nitro­gen donor with a carboxyl­ate oxygen donor site, enabling N,O-chelation toward metal centres. Such N,O-chelating systems are widely encountered in coordination chemistry and are known to stabilize a variety of metal ions and coordination geometries (Constable, 2008 ▸; Aromí *et al.*, 2012 ▸). In addition, the aromatic quinoline framework may participate in supra­molecular π–π stacking inter­actions, which can influence crystal packing.

Organotin(IV) compounds containing carboxyl­ate ligands exhibit significant structural diversity (Ingham *et al.*, 1960 ▸), with coordination numbers typically ranging from four to six, depending on ligand binding modes and reaction conditions (Ariza-Roldán *et al.*, 2023 ▸; Tiekink, 1991 ▸). Carboxyl­ate ligands can adopt various coordination modes (monodentate, bidentate chelating, bridging), leading to discrete mol­ecular species or extended architectures (Hulushe *et al.*, 2024 ▸; Murali *et al.*, 2023 ▸).

The combination of a quinolinium-2-carboxyl­ate ligand with a tin chloride precursor may therefore give rise to hybrid systems in which metal coordination and inter­molecular inter­actions coexist within the same structure. The present study reports the synthesis and structural characterization of such a compound.
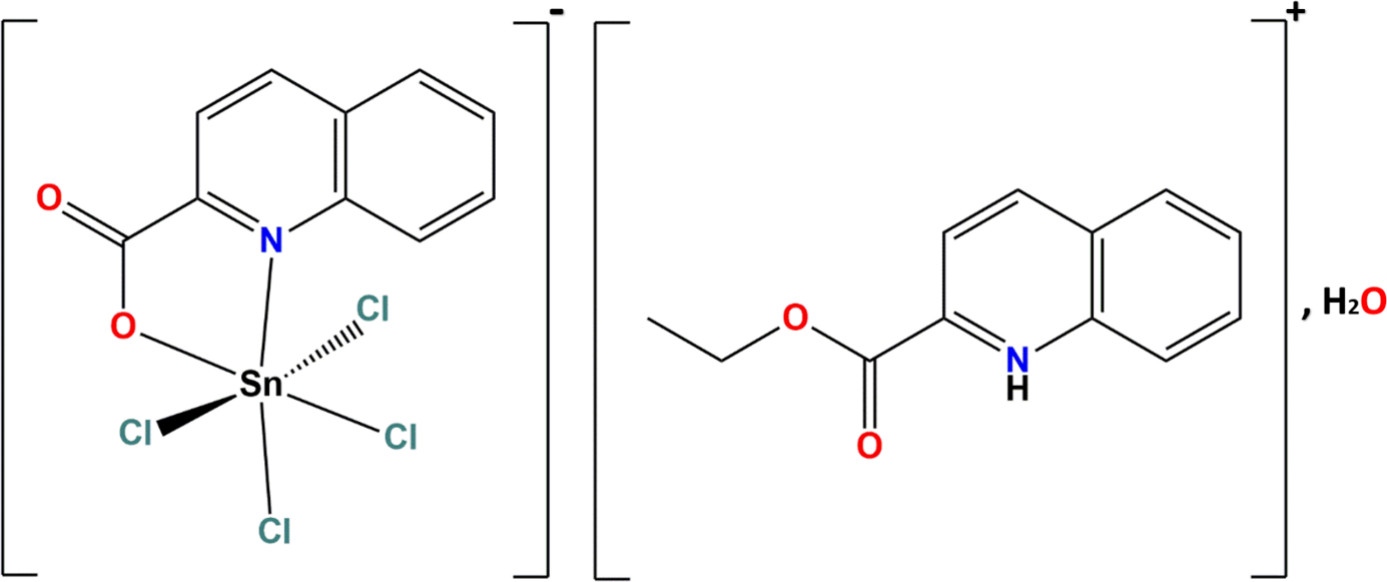


## 2. Structural commentary

**Figure 1:**
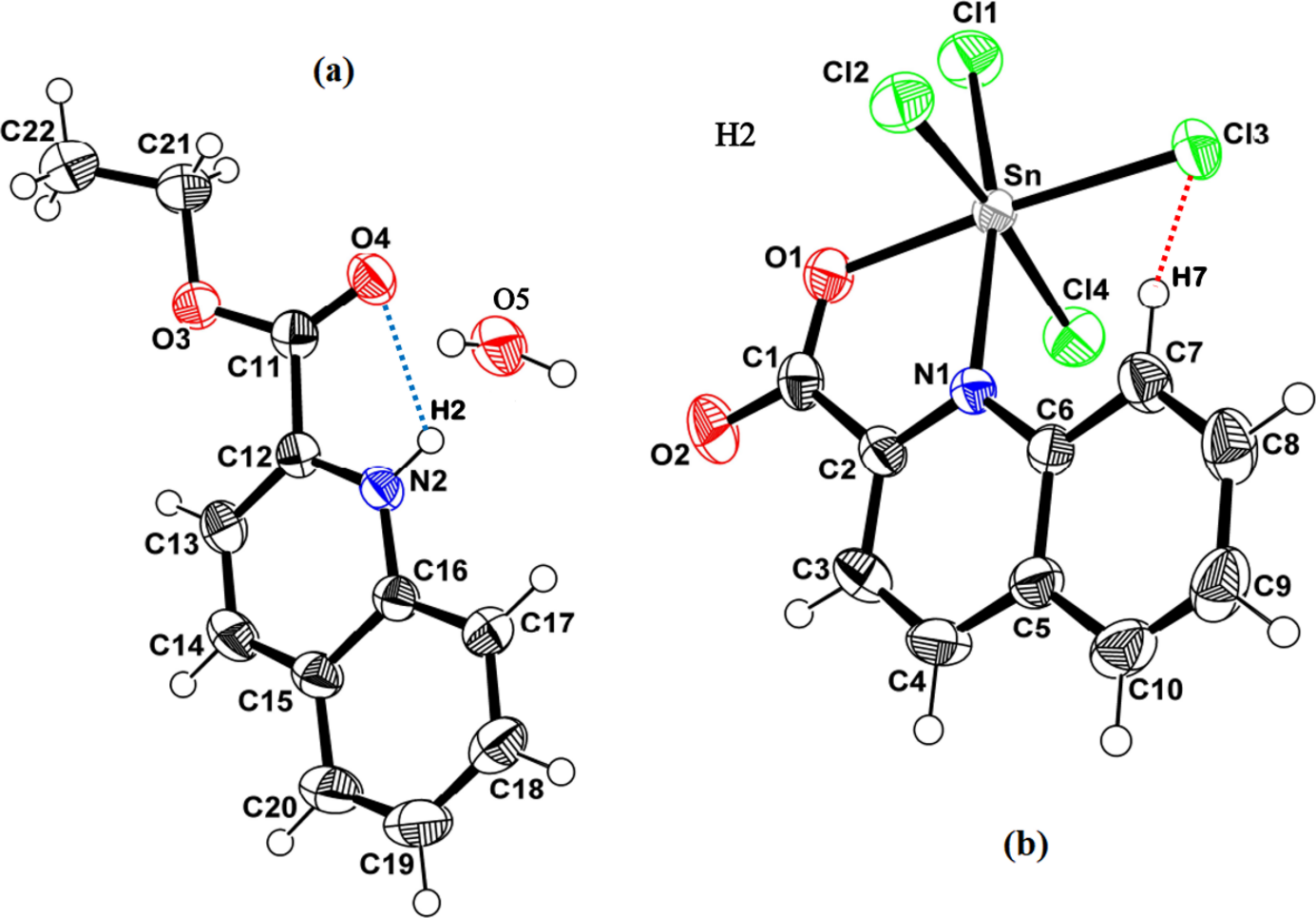
Mol­ecular view of the asymmetric unit showing: (*a*) the cationic component and the water mol­ecule, (*b*) the anionic component. Intra­molecular hydrogen bonds involving the quinolinium N—H donor and carboxyl­ate O acceptor, as well as weak C—H⋯Cl contacts are shown as dashed lines. Displacement ellipsoids are drawn at the 50% probability level.

The title salt (Fig. 1 ▸) crystallizes in the monoclinic space group *P*2_1_/*n* and is composed of a tetra­chlorido­(*N*,*O*-chelated quinolinium-2-carboxyl­atostannate(IV) anion, a protonated 2-(eth­oxy­carbon­yl)quinolinium cation and one water mol­ecule.

In the anion (Fig. 1 ▸*b*), the Sn^IV^ center shows a distorted octa­hedral coordination environment formed by four chloride ligands and by the N and O donor atoms of the quinolinium-2-carboxyl­ate ligand (Table 1 ▸). The most obvious source of distortion is the bite of the chelate: the O1—Sn1—N1 angle is only 75.87 (7)°, whereas the *trans* arrangement O1—Sn1—Cl3 is almost linear [176.38 (5)°]. The Sn—Cl distances are slightly spread [2.3779 (7)–2.4152 (7) Å], consistent with a non-regular octa­hedron, while the Sn—O and Sn—N bonds [2.0912 (17) and 2.2959 (18) Å, respectively] match well with coordination by a carboxyl­ate oxygen and a quinoline nitro­gen.

The cation (Fig. 1 ▸*a*) is a protonated quinolinium species (N2–H2) bearing an eth­oxy­carbonyl substituent. The ester group displays the expected bond-length pattern, with a short carbonyl C=O bond [O4=C11 = 1.195 (3) Å] and a longer single C—O bond [O3—C11 = 1.323 (3) Å]. The eth­oxy fragment is attached through O3—C21 [1.474 (3) Å] and adopts a common extended conformation [C11—O3—C21—C22 = −172.0 (3)°]. The crystal structure also contains a water mol­ecule, which acts as a potential hydrogen-bond donor in the subsequent supra­molecular assembly. Intra­molecular contacts include N2—H2⋯O4 and C7—H7⋯Cl3, with H⋯*A* separations of 2.41 and 2.61 Å, respectively (Fig. 1 ▸, Table 2 ▸).

## 3. Supra­molecular features

The crystal structure exhibits a well-defined supra­molecular arrangement consolidated mainly by O—H⋯O and N—H⋯O hydrogen bonds, together with weaker C—H⋯O, O—H⋯Cl and C—H⋯Cl inter­actions (Table 2 ▸). These contacts organize the components into a chain motif that propagates along the *b*-axis direction.

**Figure 2:**
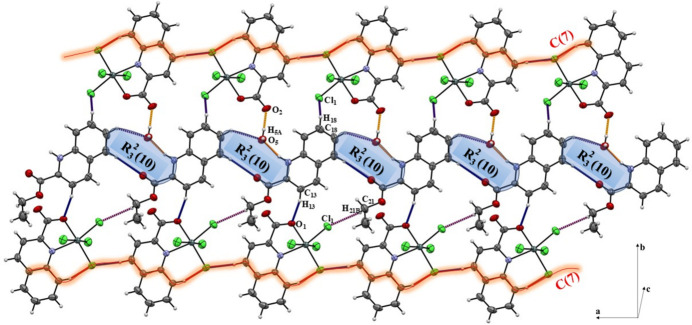
Crystal packing viewed along the *c* axis, showing the formation of hydrogen-bonded chains generated by N—H⋯O and C—H⋯O hydrogen bonds forming an 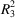
(10) ring motif: Short, inter­mediate and long hydrogen bonds are colored yellow, red and blue, respectively.

In the crystal, the water mol­ecule (O5) plays a central role in the hydrogen-bonding scheme (Table 2 ▸). The O5—H5*A·*··O2 and N2—H2⋯O5 inter­actions (H⋯*A* = 1.90 and 1.98 Å, respectively) generate 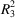
(10) ring motifs (Etter *et al.*, 1990 ▸), which link adjacent cations and anions. These rings are repeated along the *b*-axis direction, forming a continuous hydrogen-bonded chain (Fig. 2 ▸).

Additional weaker contacts, namely O5—H5*B*⋯Cl4 and C21—H21*B*⋯Cl1, give rise to *C*(7) chains that extend parallel to the *a* axis, as highlighted in Fig. 2 ▸. The C19—H19⋯O5 inter­action further reinforces the chain arrangement.

The overall packing is therefore constructed from alternating 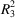
(10) ring motifs and *C*(7) chain segments, producing a layered arrangement parallel to the *ab* plane.

**Figure 3:**
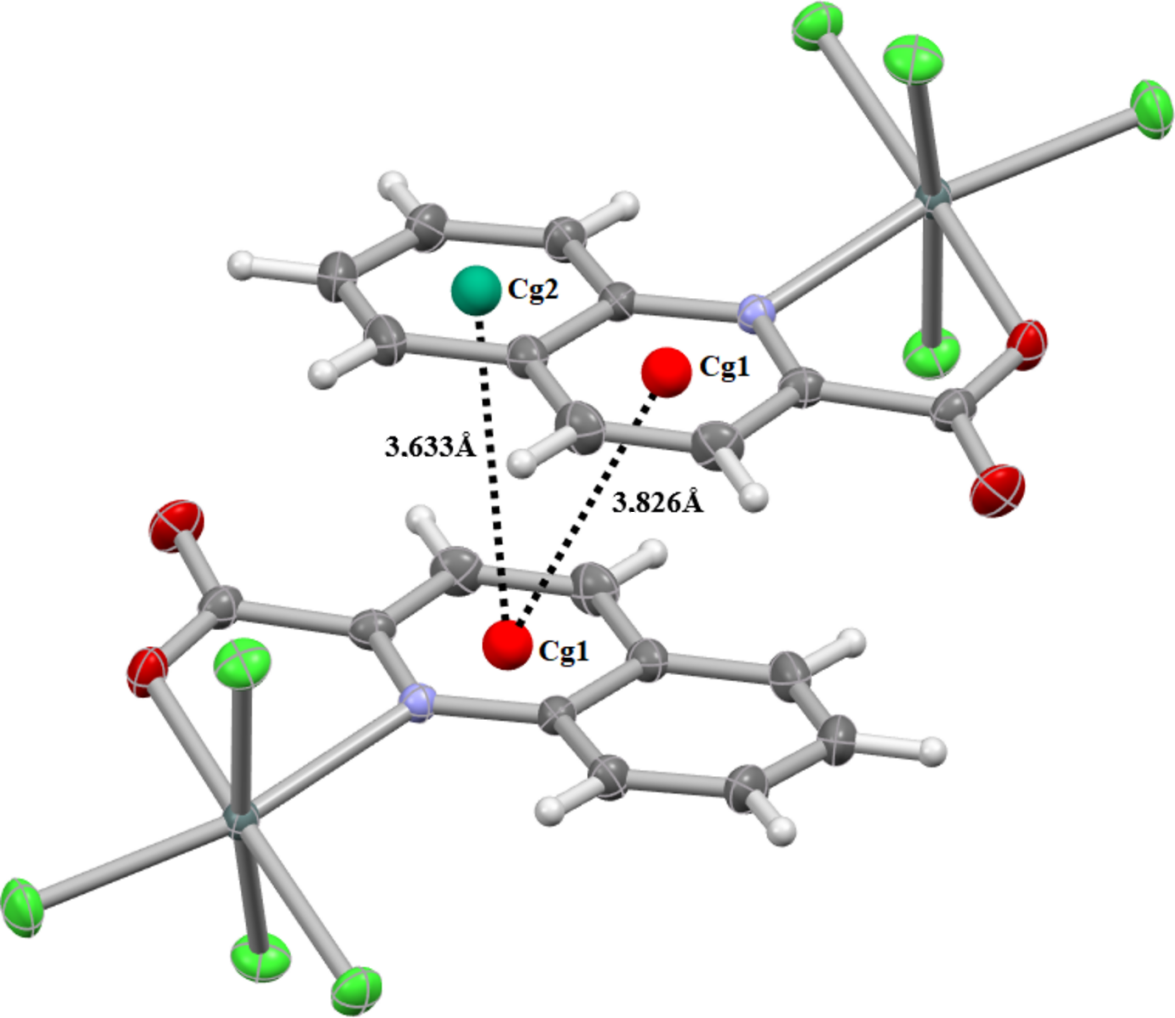
Illustration of π–π stacking inter­actions between aromatic rings of the anionic tin(IV) complex. Centroid–centroid distances (*Cg*1⋯*Cg*2/*Cg*1⋯*Cg*1) are indicated, highlighting the role of aromatic stacking in the crystal stabilization.

**Figure 4:**
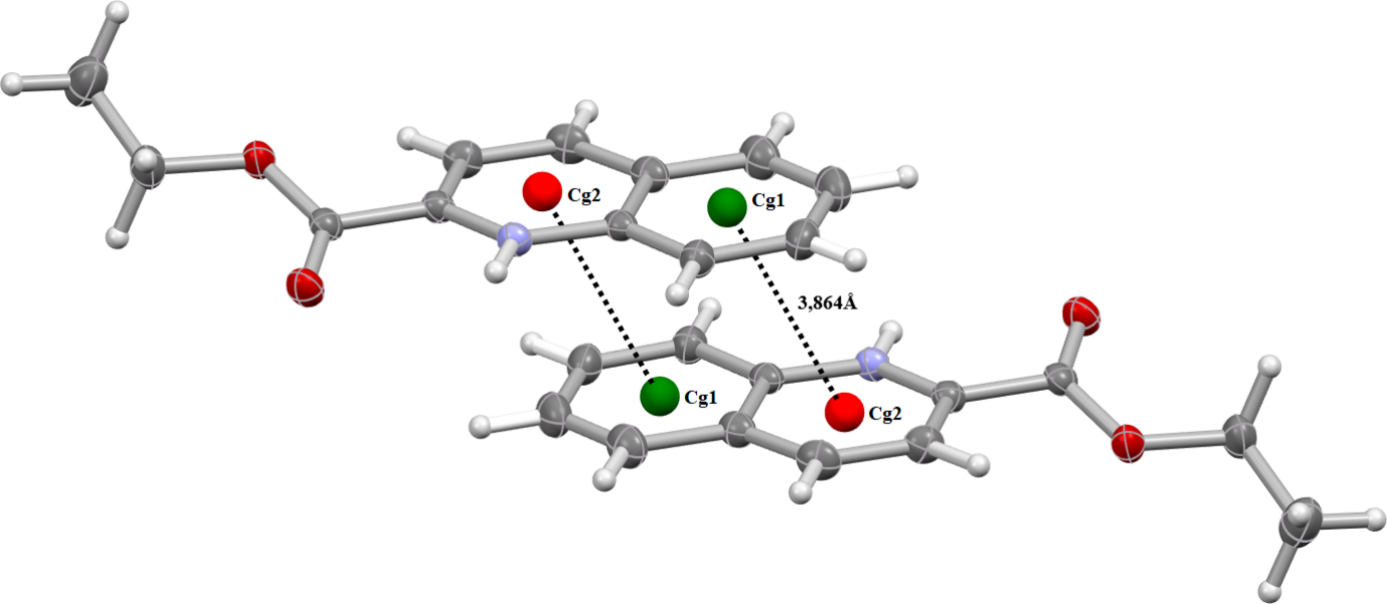
Illustration of π–π stacking inter­actions between aromatic rings of the quinolinium cation Centroid–centroid distances (*Cg*1⋯*Cg*2) are indicated by black dotted lines.

The three-dimensional framework is further supported by π–π stacking inter­actions. Within the anionic units, centroid–centroid separations of *Cg1*⋯*Cg2* = 3.633 (2) and *Cg1*⋯*Cg1* = 3.826 (2) Å are observed, where *Cg*1 and *Cg*2 are the centroids of the N1/C2–C6 and C5–C10 rings, respectively (symmetry operation 1 − *x*, 2 − *y*, 1 − *z*, Fig. 3 ▸). Similar inter­actions occur between cationic units, with a centroid–centroid distance of 3.864 (2) Å (symmetry operation −*x*, 1 − *y*, 1 − *z*) (Fig. 4 ▸).

**Figure 5:**
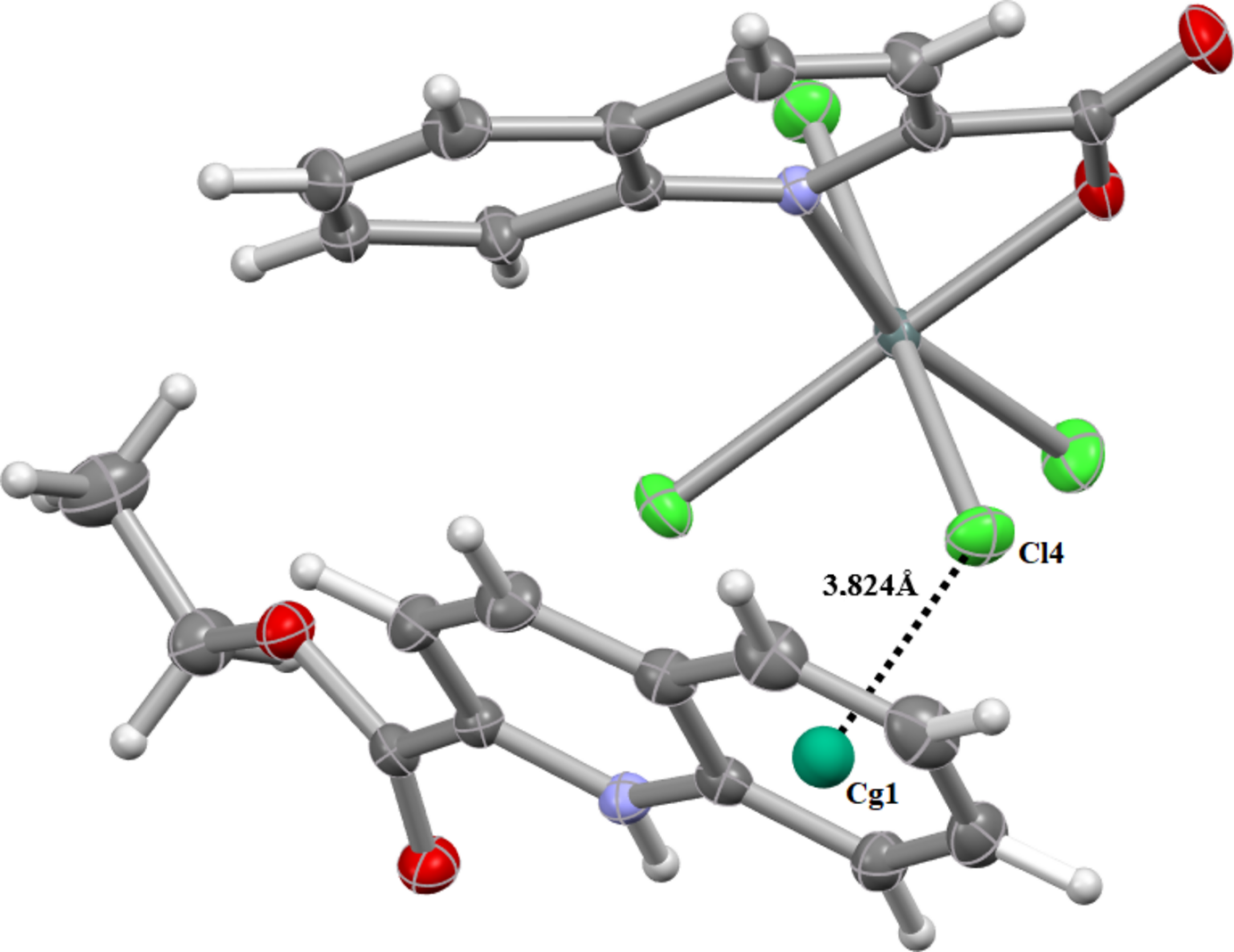
View of weak Sn—Cl⋯ π inter­actions linking neighboring cationic units, contributing to the three-dimensional supra­molecular architecture. Relevant inter­molecular distances are indicated.

In addition, an Sn1—Cl4⋯π inter­action involving the C15–C20 ring (symmetry operation 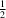 + *x*, 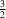 − *y*, 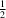 + *z*) is present, with a Cl⋯centroid separation of 3.824 (1) Å, contributing to the overall packing consolidation (Fig. 5 ▸).

## 4. Database survey

A search of the Cambridge Structural Database (CSD, version 2025.3.1, update of November 2025; Groom *et al.*, 2016 ▸) for similar compounds was undertaken.

NIPBUN (Benlatreche, 2023 ▸) crystallizes in the *C*2/*c* space group and is distinguished from the title compound by the absence of a water mol­ecule and the substitution of the ethyl group with a hydrogen atom. PAYGAZ (Najafi *et al.*, 2012 ▸) adopts the *P*2_1_/*c* space group. Its structure differs from that of the title compound by the absence of a water mol­ecule in the asymmetric unit and by the replacement of the ethyl group with an isopropyl group. TITNEQ (Wang *et al.*, 2008 ▸) crystallizes in the same space group as PAYGAZ and contains the same cation as the title compound; the main difference is presence of a butyl substituent replacing a Cl atom of the anion.

AYISUX (Najafi *et al.*, 2011 ▸) crystallizes in the *P*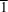 space group. While it contains the same anion as the title compound, it differs by the presence of a 4-(di­methyl­amino)­pyridinium cation instead of the original cation and by the absence of the water mol­ecule in the crystal structure. KURQUK (Vafaee *et al.*, 2010 ▸) exhibits a structural arrangement similar to that of the title compound. It differs, however, by the presence of a methanol mol­ecule in place of the water mol­ecule, as well as by the replacement of the ethyl group with a methyl group.

## 5. Hirshfeld surface analysis

**Figure 6:**
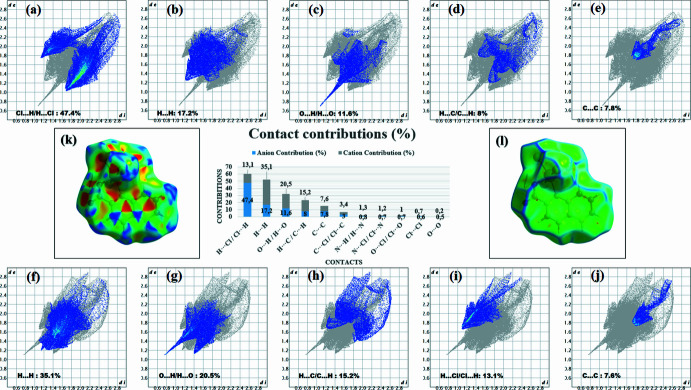
Hirshfeld surfaces mapped over *d_norm_* and corresponding two-dimensional fingerprint plots of the title compound. For the anion: (*a*) Cl⋯H/H⋯Cl, (*b*) H⋯H, (*c*) O⋯H/H⋯O, (*d*) C⋯H/H⋯C, and (*e*) C⋯C contacts. For the cation: (*f*) H⋯H, (*g*) O⋯H/H⋯O, (*h*) C⋯H/H⋯C, (*i*) Cl⋯H/H⋯Cl, and (*j*) C⋯C contacts. Hirshfeld surfaces mapped over (*j*) shape-index and (*k*) curvedness.

A Hirshfeld surface (HS) analysis (Spackman & Jayatilaka, 2009 ▸) was performed using *CrystalExplorer 21.5* (Spackman *et al.*, 2021 ▸) to qu­antify the inter­molecular inter­actions governing the crystal packing. The HS mapped over *d*_norm_ highlights close inter­molecular contacts through distinct red regions corresponding to O—H⋯O, N—H⋯O and C—H⋯Cl hydrogen-bonding inter­actions. Additional evidence for π–π stacking is provided by the Hirshfeld surfaces mapped over shape-index (Fig. 6 ▸*k*) and curvedness (Fig. 6 ▸*l*).

For the anion, the two-dimensional fingerprint plots reveal that H⋯Cl/Cl⋯H (Fig. 6 ▸*a*) contacts give the dominant contribution (47.4%), reflecting the prevalence of C—H⋯Cl hydrogen bonds in the packing. H⋯H contacts (Fig. 6 ▸*b*) account for 17.2%, indicating significant dispersive inter­actions, while O⋯H/H⋯O contacts (Fig. 6 ▸*c*) contribute 11.6%, consistent with hydrogen bonding involving the carboxyl­ate oxygen atoms. The H⋯C/C⋯H contacts (Fig. 6 ▸*d*) (8.0%) correspond to C—H⋯π hydrogen bonds, and the C⋯C contacts (Fig. 6 ▸*e*, 7.8%) are indicative of π–π stacking inter­actions. Minor contributions arise from C⋯Cl/Cl⋯C contacts (3%) and other contacts below 1%.

For the cation, the Hirshfeld surface is dominated by H⋯H contacts (35.1%, Fig. 6 ▸*f*), highlighting the importance of dispersive inter­actions. O⋯H/H⋯O contacts (Fig. 6 ▸*g*) represent 20.5% of the surface area and are attributable to N—H⋯O and C—H⋯O hydrogen bonds. The H⋯C/C⋯H contacts (Fig. 6 ▸*h*) contribute 15.2%, consistent with C—H⋯π inter­actions, while H⋯Cl/Cl⋯H contacts (Fig. 6 ▸*i*) account for 13.1%. The C⋯C contacts (7.6%, Fig. 6 ▸*j*) confirm the presence of π–π stacking inter­actions, whereas C⋯Cl/Cl⋯C contacts (3.4%) and other minor contacts contribute only marginally.

Overall, the combined analysis of the Hirshfeld surface mapped over *d*_norm_, shape-index and curvedness and the fingerprint plots demonstrate that the crystal packing is governed by a balance between hydrogen bonding, halogen-involving contacts, π–π stacking inter­actions and dispersive forces.

## 6. Synthesis and crystallization

The compound was prepared by refluxing for 6 h a solution of tin(II) chloride dihydrate (0.113 g, 0.5 mmol) in ethanol (25 mL) with quinaldic acid (0.086 g, 0.5 mmol) dissolved in the same solvent. A few drops of concentrated hydro­chloric acid were added to the reaction mixture. The resulting white solid was collected by filtration. The oxidation of Sn^II^ to Sn^IV^ most likely occurred during reflux in air.

Colorless crystals suitable for X-ray diffraction analysis were obtained by slow crystallization of the filtrate from acetone at room temperature over seven days. Yield: 87%.

IR (KBr, cm^−1^): 3454 (O—H), 3135 (C—H), 1623 (C=N), 1540 (C=C), 1484–1457 (C—H), 1357 (COO), 1310 (C—O), 590 (Sn—O), 470 (Sn—Cl).

## 7. Refinement

Crystal data, data collection and structure refinement details are summarized in Table 3 ▸. C-bound H atoms were placed geometrically and refined as riding atoms [C—H = 0.95–0.99 Å and *U*_iso_(H) = 1.2*U*_eq_(C)]. The hydrogen atoms attached to nitro­gen and oxygen were located in difference-Fourier maps and refined with distance restraints (N—H = 0.88 Å, O—H = 0.87 Å), with *U*_iso_(H) set to 1.2*U*_eq_(N) and 1.5*U*_eq_(O).

## Supplementary Material

Crystal structure: contains datablock(s) I. DOI: 10.1107/S2056989026003932/zn2048sup1.cif

Structure factors: contains datablock(s) I. DOI: 10.1107/S2056989026003932/zn2048Isup2.hkl

CCDC reference: 2269813

Additional supporting information:  crystallographic information; 3D view; checkCIF report

## Figures and Tables

**Table 1:** Selected geometric parameters (Å, °)

Sn1—Cl3	2.3919 (6)	O1—C1	1.280 (3)
Sn1—Cl4	2.4152 (7)	O3—C11	1.323 (3)
Sn1—Cl2	2.3779 (7)	O3—C21	1.474 (3)
Sn1—Cl1	2.3796 (7)	O4—C11	1.195 (3)
Sn1—O1	2.0912 (17)	O2—C1	1.225 (3)
Sn1—N1	2.2959 (18)		
			
O1—Sn1—Cl3	176.38 (5)	O1—Sn1—N1	75.87 (7)
			
C11—O3—C21—C22	−172.0 (3)		

**Table 2:** Hydrogen-bond geometry (Å, °)

*D*—H⋯*A*	*D*—H	H⋯*A*	*D*⋯*A*	*D*—H⋯*A*
N2—H2⋯O4	0.88	2.41	2.746 (3)	103
N2—H2⋯O5	0.88	1.98	2.772 (3)	149
O5—H5*A*⋯O2	0.87	1.90	2.769 (3)	173
O5—H5*B*⋯Cl4^i^	0.87	2.78	3.457 (2)	136
C7—H7⋯Cl3	0.95	2.61	3.370 (3)	137
C19—H19⋯O5^ii^	0.95	2.56	3.303 (3)	135
C21—H21*B*⋯Cl1^iii^	0.99	2.82	3.433 (3)	121
C13—H13⋯O1^iv^	0.95	2.86	3.449 (3)	121
C18—H18⋯Cl1^v^	0.95	2.92	3.601 (3)	130

Symmetry codes: (i) 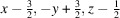; (ii) 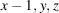; (iii) 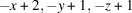; (iv) 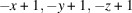; (v) 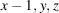.

**Table 3:** Experimental details

Crystal data	
Chemical formula	(C_12_H_12_NO_2_)[Sn(C_10_H_6_NO_2_)Cl_4_]·H_2_O
*M*_r_	652.89
Crystal system, space group	Monoclinic, *P*2_1_/*n*
Temperature (K)	150
*a*, *b*, *c* (Å)	9.0191 (4), 17.3856 (9), 16.0742 (7)
β (°)	95.063 (3)
*V* (Å^3^)	2510.6 (2)
*Z*	4
Radiation type	Mo *K*α
μ (mm^−1^)	1.48
Crystal size (mm)	0.41 × 0.35 × 0.21

Data collection	
Diffractometer	D8 VENTURE Bruker AXS
Absorption correction	Multi-scan (*SADABS*; Krause *et al.*, 2015 ▸)
No. of measured, independent and observed [*I* > 2σ(*I*)] reflections	36638, 5735, 5120
*R*_int_	0.037
(sin θ/λ)_max_ (Å^−1^)	0.650

Refinement	
*R*[*F*^2^ > 2σ(*F*^2^)], *wR*(*F*^2^), *S*	0.027, 0.059, 1.08
No. of reflections	5735
No. of parameters	312
H-atom treatment	H-atom parameters constrained
Δρ_max_, Δρ_min_ (e Å^−3^)	0.37, −0.36

Computer programs: *APEX3* and *SAINT* (Bruker, 2015 ▸), *SHELXT2018/2* (Sheldrick, 2015*a* ▸), *SHELXL2018/3* (Sheldrick, 2015*b* ▸) and *OLEX2* (Dolomanov *et al.*, 2009 ▸).

## supplementary crystallographic information

### 2-(Ethoxycarbonyl)quinolinium tetrachlorido(quinoline-2-carboxylato-κ2N,O)stannate(IV) monohydrate Crystal data

**Table:**

(C_12_H_12_NO_2_)[Sn(C_10_H_6_NO_2_)Cl_4_]·H_2_O	*F*(000) = 1296
*M**_r_* = 652.89	*D*_x_ = 1.727 Mg m^−^^3^
Monoclinic, *P*2_1_/*n*	Mo *K*α radiation, λ = 0.71073 Å
*a* = 9.0191 (4) Å	Cell parameters from 8949 reflections
*b* = 17.3856 (9) Å	θ = 2.3–27.5°
*c* = 16.0742 (7) Å	µ = 1.48 mm^−^^1^
β = 95.063 (3)°	*T* = 150 K
*V* = 2510.6 (2) Å^3^	Prism, colourless
*Z* = 4	0.41 × 0.35 × 0.21 mm

### 2-(Ethoxycarbonyl)quinolinium tetrachlorido(quinoline-2-carboxylato-κ2N,O)stannate(IV) monohydrate Data collection

**Table:**

D8 VENTURE Bruker AXS diffractometer	5120 reflections with *I* > 2σ(*I*)
Detector resolution: 10.4167 pixels mm^-1^	*R*_int_ = 0.037
rotation images scans	θ_max_ = 27.5°, θ_min_ = 2.5°
Absorption correction: multi-scan (SADABS; Krause *et al.*, 2015)	*h* = −11→11
	*k* = −22→22
36638 measured reflections	*l* = −20→20
5735 independent reflections	

### 2-(Ethoxycarbonyl)quinolinium tetrachlorido(quinoline-2-carboxylato-κ2N,O)stannate(IV) monohydrate Refinement

**Table:**

Refinement on *F*^2^	Hydrogen site location: mixed
Least-squares matrix: full	H-atom parameters constrained
*R*[*F*^2^ > 2σ(*F*^2^)] = 0.027	*w* = 1/[σ^2^(*F*_o_^2^) + (0.0057*P*)^2^ + 2.9951*P*] where *P* = (*F*_o_^2^ + 2*F*_c_^2^)/3
*wR*(*F*^2^) = 0.059	(Δ/σ)_max_ = 0.002
*S* = 1.08	Δρ_max_ = 0.37 e Å^−^^3^
5735 reflections	Δρ_min_ = −0.36 e Å^−^^3^
312 parameters	Extinction correction: SHELXL2018/3 (Sheldrick 2015b), Fc^*^=kFc[1+0.001xFc^2^λ^3^/sin(2θ)]^-/4^
0 restraints	Extinction coefficient: 0.0039 (2)
Primary atom site location: dual	

### 2-(Ethoxycarbonyl)quinolinium tetrachlorido(quinoline-2-carboxylato-κ2N,O)stannate(IV) monohydrate Special details

**Table:**

Geometry. All esds (except the esd in the dihedral angle between two l.s. planes) are estimated using the full covariance matrix. The cell esds are taken into account individually in the estimation of esds in distances, angles and torsion angles; correlations between esds in cell parameters are only used when they are defined by crystal symmetry. An approximate (isotropic) treatment of cell esds is used for estimating esds involving l.s. planes.

### 2-(Ethoxycarbonyl)quinolinium tetrachlorido(quinoline-2-carboxylato-κ2N,O)stannate(IV) monohydrate Fractional atomic coordinates and isotropic or equivalent isotropic displacement parameters (Å^2^)

**Table:**

	*x*	*y*	*z*	*U*_iso_*/*U*_eq_	
Sn1	0.87992 (2)	0.85623 (2)	0.63873 (2)	0.02860 (6)	
Cl3	1.02063 (7)	0.95405 (4)	0.71472 (4)	0.04512 (16)	
Cl4	0.76618 (8)	0.82289 (4)	0.76437 (4)	0.04795 (16)	
Cl2	0.96047 (8)	0.89570 (4)	0.50842 (4)	0.04680 (16)	
Cl1	1.06888 (8)	0.76150 (4)	0.66430 (5)	0.05258 (18)	
O1	0.74573 (19)	0.77227 (9)	0.57682 (12)	0.0390 (4)	
O3	0.4585 (2)	0.36787 (10)	0.30635 (11)	0.0416 (4)	
O4	0.5664 (2)	0.44352 (11)	0.40783 (12)	0.0440 (4)	
N1	0.6575 (2)	0.91778 (10)	0.60494 (11)	0.0274 (4)	
O2	0.5144 (2)	0.73894 (11)	0.53402 (13)	0.0518 (5)	
O5	0.5333 (2)	0.62082 (12)	0.41992 (14)	0.0521 (5)	
H5A	0.525233	0.660513	0.452171	0.078*	
H5B	0.516495	0.638959	0.369527	0.078*	
N2	0.2983 (2)	0.51761 (11)	0.41244 (12)	0.0321 (4)	
H2	0.382278	0.539846	0.431233	0.039*	
C6	0.6161 (3)	0.99322 (13)	0.61617 (14)	0.0294 (5)	
C16	0.1678 (3)	0.55499 (14)	0.42247 (14)	0.0321 (5)	
C7	0.7236 (3)	1.05195 (14)	0.62800 (16)	0.0383 (5)	
H7	0.826336	1.039946	0.628147	0.046*	
C11	0.4595 (3)	0.41997 (13)	0.36641 (15)	0.0343 (5)	
C2	0.5530 (3)	0.86531 (14)	0.58426 (15)	0.0324 (5)	
C13	0.1751 (3)	0.41027 (15)	0.34784 (16)	0.0392 (6)	
H13	0.179367	0.360714	0.323268	0.047*	
C15	0.0326 (3)	0.51842 (15)	0.39287 (16)	0.0378 (5)	
C12	0.3047 (3)	0.44922 (13)	0.37563 (14)	0.0315 (5)	
C5	0.4623 (3)	1.01320 (14)	0.61270 (15)	0.0352 (5)	
C1	0.6060 (3)	0.78562 (14)	0.56387 (15)	0.0358 (5)	
C4	0.3560 (3)	0.95500 (17)	0.59421 (18)	0.0451 (6)	
H4	0.252904	0.966580	0.592848	0.054*	
C17	0.1682 (3)	0.62822 (15)	0.45981 (17)	0.0399 (6)	
H17	0.258951	0.651817	0.480620	0.048*	
C20	−0.1023 (3)	0.55863 (18)	0.4016 (2)	0.0507 (7)	
H20	−0.194869	0.535698	0.382773	0.061*	
C8	0.6796 (3)	1.12639 (15)	0.63930 (19)	0.0478 (7)	
H8	0.752851	1.165614	0.647242	0.057*	
C14	0.0409 (3)	0.44497 (16)	0.35670 (18)	0.0448 (6)	
H14	−0.048396	0.418874	0.338021	0.054*	
C3	0.4009 (3)	0.88209 (16)	0.57827 (18)	0.0424 (6)	
H3	0.329691	0.843117	0.563270	0.051*	
C21	0.6063 (3)	0.33869 (19)	0.2895 (2)	0.0525 (7)	
H21A	0.649600	0.308108	0.337666	0.063*	
H21B	0.674041	0.382200	0.280822	0.063*	
C18	0.0352 (3)	0.66467 (17)	0.46549 (18)	0.0487 (7)	
H18	0.033961	0.714599	0.489433	0.058*	
C19	−0.0994 (3)	0.6296 (2)	0.4366 (2)	0.0553 (8)	
H19	−0.190331	0.656018	0.441600	0.066*	
C10	0.4233 (3)	1.09112 (17)	0.62584 (18)	0.0483 (7)	
H10	0.321222	1.104909	0.625091	0.058*	
C9	0.5285 (4)	1.14585 (16)	0.63936 (18)	0.0497 (7)	
H9	0.500479	1.197558	0.648930	0.060*	
C22	0.5889 (4)	0.2902 (2)	0.2140 (2)	0.0696 (10)	
H22A	0.544989	0.320784	0.166897	0.104*	
H22B	0.523579	0.246684	0.223677	0.104*	
H22C	0.686556	0.271021	0.201381	0.104*	

### 2-(Ethoxycarbonyl)quinolinium tetrachlorido(quinoline-2-carboxylato-κ2N,O)stannate(IV) monohydrate Atomic displacement parameters (Å^2^)

**Table:**

	*U*^11^	*U*^22^	*U*^33^	*U*^12^	*U*^13^	*U*^23^
Sn1	0.02830 (9)	0.02496 (8)	0.03243 (9)	0.00056 (6)	0.00209 (6)	−0.00335 (6)
Cl3	0.0418 (3)	0.0414 (3)	0.0508 (4)	−0.0070 (3)	−0.0037 (3)	−0.0130 (3)
Cl4	0.0506 (4)	0.0566 (4)	0.0376 (3)	−0.0054 (3)	0.0088 (3)	0.0084 (3)
Cl2	0.0480 (4)	0.0535 (4)	0.0406 (3)	−0.0047 (3)	0.0130 (3)	0.0017 (3)
Cl1	0.0433 (4)	0.0426 (4)	0.0705 (5)	0.0157 (3)	−0.0028 (3)	−0.0019 (3)
O1	0.0393 (10)	0.0276 (8)	0.0496 (10)	−0.0009 (7)	0.0020 (8)	−0.0086 (7)
O3	0.0388 (10)	0.0415 (10)	0.0431 (10)	0.0099 (8)	−0.0031 (8)	−0.0083 (8)
O4	0.0381 (10)	0.0443 (10)	0.0474 (11)	0.0042 (8)	−0.0085 (8)	−0.0068 (8)
N1	0.0269 (9)	0.0265 (9)	0.0287 (9)	−0.0007 (7)	0.0020 (7)	−0.0015 (7)
O2	0.0557 (12)	0.0370 (10)	0.0605 (13)	−0.0160 (9)	−0.0070 (10)	−0.0102 (9)
O5	0.0466 (11)	0.0487 (11)	0.0603 (13)	−0.0085 (9)	0.0004 (10)	−0.0108 (10)
N2	0.0284 (10)	0.0328 (10)	0.0344 (10)	−0.0029 (8)	−0.0013 (8)	−0.0021 (8)
C6	0.0323 (11)	0.0277 (11)	0.0275 (11)	0.0022 (9)	−0.0004 (9)	−0.0019 (9)
C16	0.0305 (11)	0.0378 (12)	0.0281 (11)	0.0002 (9)	0.0024 (9)	0.0040 (9)
C7	0.0374 (13)	0.0305 (12)	0.0453 (14)	−0.0010 (10)	−0.0057 (11)	−0.0026 (10)
C11	0.0384 (13)	0.0302 (12)	0.0334 (12)	0.0048 (10)	−0.0012 (10)	0.0037 (9)
C2	0.0315 (12)	0.0346 (12)	0.0306 (11)	−0.0057 (9)	0.0008 (9)	−0.0003 (9)
C13	0.0408 (14)	0.0338 (13)	0.0422 (14)	−0.0076 (10)	−0.0005 (11)	−0.0034 (10)
C15	0.0302 (12)	0.0453 (14)	0.0378 (13)	−0.0031 (10)	0.0030 (10)	0.0046 (11)
C12	0.0348 (12)	0.0281 (11)	0.0312 (12)	0.0008 (9)	0.0000 (9)	0.0008 (9)
C5	0.0340 (12)	0.0392 (13)	0.0322 (12)	0.0059 (10)	0.0023 (10)	−0.0013 (10)
C1	0.0437 (14)	0.0291 (12)	0.0342 (12)	−0.0068 (10)	0.0018 (10)	−0.0025 (10)
C4	0.0279 (12)	0.0541 (16)	0.0532 (16)	0.0044 (11)	0.0039 (11)	0.0002 (13)
C17	0.0401 (14)	0.0391 (14)	0.0408 (14)	0.0037 (11)	0.0047 (11)	−0.0036 (11)
C20	0.0332 (14)	0.0622 (19)	0.0569 (18)	−0.0006 (13)	0.0056 (12)	0.0051 (15)
C8	0.0598 (18)	0.0290 (13)	0.0516 (16)	−0.0003 (12)	−0.0106 (14)	−0.0052 (11)
C14	0.0355 (14)	0.0487 (15)	0.0491 (16)	−0.0127 (12)	−0.0029 (12)	−0.0009 (12)
C3	0.0302 (12)	0.0456 (14)	0.0505 (16)	−0.0090 (11)	−0.0003 (11)	0.0000 (12)
C21	0.0401 (15)	0.0610 (19)	0.0556 (18)	0.0146 (13)	0.0004 (13)	−0.0106 (14)
C18	0.0551 (17)	0.0471 (16)	0.0452 (15)	0.0120 (13)	0.0114 (13)	−0.0033 (12)
C19	0.0400 (15)	0.070 (2)	0.0577 (18)	0.0152 (14)	0.0123 (14)	0.0062 (15)
C10	0.0485 (16)	0.0501 (16)	0.0455 (15)	0.0212 (13)	0.0003 (12)	−0.0033 (13)
C9	0.0653 (19)	0.0350 (14)	0.0473 (16)	0.0158 (13)	−0.0049 (14)	−0.0078 (12)
C22	0.0492 (18)	0.094 (3)	0.067 (2)	0.0049 (18)	0.0096 (16)	−0.024 (2)

### 2-(Ethoxycarbonyl)quinolinium tetrachlorido(quinoline-2-carboxylato-κ2N,O)stannate(IV) monohydrate Geometric parameters (Å, º)

**Table:**

Sn1—Cl3	2.3919 (6)	C13—C12	1.390 (3)
Sn1—Cl4	2.4152 (7)	C13—C14	1.370 (4)
Sn1—Cl2	2.3779 (7)	C15—C20	1.421 (4)
Sn1—Cl1	2.3796 (7)	C15—C14	1.408 (4)
Sn1—O1	2.0912 (17)	C5—C4	1.407 (4)
Sn1—N1	2.2959 (18)	C5—C10	1.420 (4)
O1—C1	1.280 (3)	C4—H4	0.9500
O3—C11	1.323 (3)	C4—C3	1.362 (4)
O3—C21	1.474 (3)	C17—H17	0.9500
O4—C11	1.195 (3)	C17—C18	1.367 (4)
N1—C6	1.380 (3)	C20—H20	0.9500
N1—C2	1.333 (3)	C20—C19	1.355 (4)
O2—C1	1.225 (3)	C8—H8	0.9500
O5—H5A	0.8700	C8—C9	1.404 (4)
O5—H5B	0.8700	C14—H14	0.9500
N2—H2	0.8800	C3—H3	0.9500
N2—C16	1.366 (3)	C21—H21A	0.9900
N2—C12	1.332 (3)	C21—H21B	0.9900
C6—C7	1.409 (3)	C21—C22	1.474 (4)
C6—C5	1.426 (3)	C18—H18	0.9500
C16—C15	1.419 (3)	C18—C19	1.400 (5)
C16—C17	1.407 (3)	C19—H19	0.9500
C7—H7	0.9500	C10—H10	0.9500
C7—C8	1.370 (3)	C10—C9	1.348 (4)
C11—C12	1.506 (3)	C9—H9	0.9500
C2—C1	1.511 (3)	C22—H22A	0.9800
C2—C3	1.397 (3)	C22—H22B	0.9800
C13—H13	0.9500	C22—H22C	0.9800
			
Cl3—Sn1—Cl4	89.37 (3)	C4—C5—C6	118.3 (2)
Cl2—Sn1—Cl3	93.16 (3)	C4—C5—C10	123.0 (2)
Cl2—Sn1—Cl4	172.39 (3)	C10—C5—C6	118.7 (2)
Cl2—Sn1—Cl1	94.71 (3)	O1—C1—C2	117.2 (2)
Cl1—Sn1—Cl3	93.68 (3)	O2—C1—O1	124.3 (2)
Cl1—Sn1—Cl4	92.29 (3)	O2—C1—C2	118.5 (2)
O1—Sn1—Cl3	176.38 (5)	C5—C4—H4	120.0
O1—Sn1—Cl4	87.69 (5)	C3—C4—C5	120.1 (2)
O1—Sn1—Cl2	89.50 (5)	C3—C4—H4	120.0
O1—Sn1—Cl1	88.55 (5)	C16—C17—H17	120.7
O1—Sn1—N1	75.87 (7)	C18—C17—C16	118.6 (3)
N1—Sn1—Cl3	101.68 (5)	C18—C17—H17	120.7
N1—Sn1—Cl4	83.28 (5)	C15—C20—H20	119.9
N1—Sn1—Cl2	89.17 (5)	C19—C20—C15	120.2 (3)
N1—Sn1—Cl1	163.93 (5)	C19—C20—H20	119.9
C1—O1—Sn1	118.09 (15)	C7—C8—H8	119.3
C11—O3—C21	114.9 (2)	C7—C8—C9	121.3 (3)
C6—N1—Sn1	130.78 (15)	C9—C8—H8	119.3
C2—N1—Sn1	108.91 (15)	C13—C14—C15	121.5 (2)
C2—N1—C6	119.29 (19)	C13—C14—H14	119.3
H5A—O5—H5B	104.5	C15—C14—H14	119.3
C16—N2—H2	118.4	C2—C3—H3	120.4
C12—N2—H2	118.4	C4—C3—C2	119.2 (2)
C12—N2—C16	123.3 (2)	C4—C3—H3	120.4
N1—C6—C7	121.0 (2)	O3—C21—H21A	110.0
N1—C6—C5	120.0 (2)	O3—C21—H21B	110.0
C7—C6—C5	118.9 (2)	O3—C21—C22	108.4 (2)
N2—C16—C15	118.1 (2)	H21A—C21—H21B	108.4
N2—C16—C17	120.7 (2)	C22—C21—H21A	110.0
C17—C16—C15	121.2 (2)	C22—C21—H21B	110.0
C6—C7—H7	120.0	C17—C18—H18	119.5
C8—C7—C6	119.9 (2)	C17—C18—C19	121.0 (3)
C8—C7—H7	120.0	C19—C18—H18	119.5
O3—C11—C12	110.9 (2)	C20—C19—C18	121.3 (3)
O4—C11—O3	126.5 (2)	C20—C19—H19	119.4
O4—C11—C12	122.6 (2)	C18—C19—H19	119.4
N1—C2—C1	116.8 (2)	C5—C10—H10	119.4
N1—C2—C3	122.9 (2)	C9—C10—C5	121.1 (3)
C3—C2—C1	120.3 (2)	C9—C10—H10	119.4
C12—C13—H13	120.8	C8—C9—H9	120.0
C14—C13—H13	120.8	C10—C9—C8	120.0 (2)
C14—C13—C12	118.5 (2)	C10—C9—H9	120.0
C16—C15—C20	117.7 (2)	C21—C22—H22A	109.5
C14—C15—C16	118.0 (2)	C21—C22—H22B	109.5
C14—C15—C20	124.4 (3)	C21—C22—H22C	109.5
N2—C12—C11	115.0 (2)	H22A—C22—H22B	109.5
N2—C12—C13	120.6 (2)	H22A—C22—H22C	109.5
C13—C12—C11	124.4 (2)	H22B—C22—H22C	109.5
			
Sn1—O1—C1—O2	−174.3 (2)	C7—C6—C5—C4	175.0 (2)
Sn1—O1—C1—C2	7.9 (3)	C7—C6—C5—C10	−3.4 (3)
Sn1—N1—C6—C7	20.8 (3)	C7—C8—C9—C10	−1.8 (5)
Sn1—N1—C6—C5	−161.67 (16)	C11—O3—C21—C22	−172.0 (3)
Sn1—N1—C2—C1	−16.7 (2)	C2—N1—C6—C7	−172.2 (2)
Sn1—N1—C2—C3	165.6 (2)	C2—N1—C6—C5	5.4 (3)
O3—C11—C12—N2	−158.3 (2)	C15—C16—C17—C18	−1.5 (4)
O3—C11—C12—C13	20.9 (3)	C15—C20—C19—C18	−0.5 (5)
O4—C11—C12—N2	20.1 (3)	C12—N2—C16—C15	0.7 (3)
O4—C11—C12—C13	−160.7 (3)	C12—N2—C16—C17	−178.2 (2)
N1—C6—C7—C8	−179.8 (2)	C12—C13—C14—C15	0.1 (4)
N1—C6—C5—C4	−2.6 (3)	C5—C6—C7—C8	2.6 (4)
N1—C6—C5—C10	179.0 (2)	C5—C4—C3—C2	2.9 (4)
N1—C2—C1—O1	7.5 (3)	C5—C10—C9—C8	1.0 (4)
N1—C2—C1—O2	−170.4 (2)	C1—C2—C3—C4	−177.8 (2)
N1—C2—C3—C4	−0.1 (4)	C4—C5—C10—C9	−176.7 (3)
N2—C16—C15—C20	−178.3 (2)	C17—C16—C15—C20	0.6 (4)
N2—C16—C15—C14	1.5 (3)	C17—C16—C15—C14	−179.6 (2)
N2—C16—C17—C18	177.4 (2)	C17—C18—C19—C20	−0.4 (5)
C6—N1—C2—C1	173.7 (2)	C20—C15—C14—C13	178.0 (3)
C6—N1—C2—C3	−4.1 (3)	C14—C13—C12—N2	2.1 (4)
C6—C7—C8—C9	0.0 (4)	C14—C13—C12—C11	−177.0 (2)
C6—C5—C4—C3	−1.5 (4)	C14—C15—C20—C19	−179.4 (3)
C6—C5—C10—C9	1.6 (4)	C3—C2—C1—O1	−174.7 (2)
C16—N2—C12—C11	176.7 (2)	C3—C2—C1—O2	7.4 (4)
C16—N2—C12—C13	−2.6 (4)	C21—O3—C11—O4	−1.4 (4)
C16—C15—C20—C19	0.4 (4)	C21—O3—C11—C12	176.9 (2)
C16—C15—C14—C13	−1.9 (4)	C10—C5—C4—C3	176.8 (3)
C16—C17—C18—C19	1.4 (4)		

### 2-(Ethoxycarbonyl)quinolinium tetrachlorido(quinoline-2-carboxylato-κ2N,O)stannate(IV) monohydrate Hydrogen-bond geometry (Å, º)

**Table:**

*D*—H···*A*	*D*—H	H···*A*	*D*···*A*	*D*—H···*A*
N2—H2···O4	0.88	2.41	2.746 (3)	103
N2—H2···O5	0.88	1.98	2.772 (3)	149
O5—H5*A*···O2	0.87	1.90	2.769 (3)	173
O5—H5*B*···Cl4^i^	0.87	2.78	3.457 (2)	136
C7—H7···Cl3	0.95	2.61	3.370 (3)	137
C19—H19···O5^ii^	0.95	2.56	3.303 (3)	135
C21—H21*B*···Cl1^iii^	0.99	2.82	3.433 (3)	121
C13—H13···O1^iv^	0.95	2.86	3.449 (3)	121
C18—H18···Cl1^v^	0.95	2.92	3.601 (3)	130

Symmetry codes: (i) *x*−3/2, −*y*+1/2, *z*−3/2; (ii) *x*−1, *y*, *z*; (iii) −*x*+2, −*y*+1, −*z*+1; (iv) −*x*+1, −*y*+1, −*z*+1; (v) *x*+1, *y*, *z*.
